# Instruments to assess the digital health competencies of healthcare professionals: a scoping review

**DOI:** 10.3389/fpubh.2025.1726452

**Published:** 2026-01-05

**Authors:** Joana Paias Ferreira, Teresa Magalhães

**Affiliations:** 1 NOVA National School of Public Health, Public Health Research Centre, NOVA University Lisbon, Lisbon, Portugal; 2 NOVA National School of Public Health, Public Health Research Centre, Comprehensive Health Research Centre, CHRC, REAL, CCAL, NOVA University Lisbon, Lisbon, Portugal

**Keywords:** competencies, digital health, healthcare professionals, instrument, workforce development

## Abstract

**Background:**

The digital transformation of healthcare is reshaping clinical practice, healthcare management, patient-provider interactions, and traditional public health fields. These digital tools enhance safety, quality of care and efficacy, while also supporting the shift from cure to prevention, empowering patients, and promoting overall efficiency of healthcare management and delivery. In healthcare, digital competence remains inconsistently defined and its assessment is often limited by conflation with narrower constructs such as digital literacy. The aim of this scoping review is to identify instruments that capture multiple dimensions of digital health competencies, analyse their scope and reported psychometric properties. This provides a clear view of the essential digital competencies in healthcare, critical for improving patient care, development of targeted education and workforce strategies, and advancing public health functions, contributing to a more equitable and sustainable healthcare systems in the digital era.

**Methods:**

A scoping review was conducted according to the JBI Manual for Evidence Synthesis. Three databases (PubMed, Scopus, Web of Science) were searched for studies published from 2015 to June 2025, using established criteria. Identified instruments were mapped by the digital area they apply, healthcare sample and setting, dimensions of competencies assessed and number of items. Further analysis followed two frameworks for questionnaire development (ESS Handbook) and measurement properties (COSMIN).

**Results:**

Of the 441 identified studies, 19 met the inclusion criteria. Included studies predominantly used mixed professional samples; among single-group studies, nurses were most often targeted, and hospital settings dominated, with the public health field rarely represented. Most instruments adopted a generalist scope rather than focusing on a specific digital health area. Psychometric reporting was limited, with only three studies offering comprehensive psychometric validation. One instrument introduced a new perspective by explicitly assessing factors influencing the adoption of competencies.

**Conclusion:**

Interest in assessing digital health competencies has grown, and the field is continually evolving; however, psychometrically validated instruments remain scarce. Mapping multidimensional instruments reveals recurring core dimensions and adoption-related factors, providing a clear foundation for more specific, targeted, and practice-aligned education and training, and a generalizable core for a standardized, digital public health competency framework for the multi-professional public health workforce.

## 1 Introduction

Digital public health (DiPH) describes how technologies are reshaping population-level functions such as prevention, surveillance, and health promotion, and is recognised as a new but rapidly consolidating field (1, 2). In a global public health context, digital health tools can strengthen health systems, support more equitable access to care and improve the quality, efficiency and safety of healthcare by enhancing medication safety, enabling more personalised care, supporting data-driven decisions and streamlining routine operations (1, 3–7).

### 1.1 What is known

The WHO Global Strategy on Digital Health explicitly calls to “promote and facilitate digital health competencies in the education and training curricula of all health professionals and allied workers,” positioning a digitally competent workforce as a core condition for successful digital transformation of health systems (6). However, the implementation of digital health technologies in healthcare and public health still faces multiple barriers (6, 8, 9), with insufficient digital competencies among healthcare professionals highlighted as a perceived obstacle, slowing adoption, compromising quality of care, and decreasing operational effectiveness within healthcare systems (5, 9, 10). Less than 30% reporting feeling sufficiently prepared to use existing digital health tools in patient care effectively, 20% adapt them creatively to different patient needs and only 12% can identify the digital solutions currently available in their services (11).

At the same time, digital competence in healthcare is not consistently defined. It is often conflated with eHealth literacy or digital literacy, although these terms may be close, they are not synonymous, since they mainly concern the ability to search, find, understand, and evaluate digital health information (12–15). The DigComp framework conceptualizes digital competencies as a combination of knowledge, skills and attitudes for the confident, critical, and responsible use of digital technologies across multiple areas (16, 17). In healthcare, competence is similarly described as the integration of knowledge, skills, abilities, and traits needed to provide high-quality services applied to digital contexts (18), this means that digital competence goes beyond technical skills to encompass attitudes and motivation, critical appraisal of digital tools, communication in digital environments, awareness of ethical and legal issues, and adaptability to evolving technologies (3, 7, 19).

### 1.2 What remains unknown

Given this context, assessing the digital competencies of healthcare professionals becomes critically important to understand their level of digital competence and therefore to develop focused, evidence-based educational interventions tailored specifically to meet identified gaps. Ultimately facilitating a successful and sustainable digital transformation within the healthcare systems (3, 7). Although some research has been recently conducted in the digital health competencies field, no review focused solely on the instruments available to assess these competencies. Previous reviews by Longhini et al. (12) and Mainz et al. (13) have begun to summarize how these competencies have been investigated among healthcare professionals. Both reviews treated digital competence, digital literacy, and eHealth literacy together and therefore included single-dimension literacy instruments alongside broader competence measures. Additionally, a dedicated scoping review focused exclusively on instruments to assess digital health literacy and treated this term as the same as eHealth literacy (20).

### 1.3 Why mapping digital health competencies is needed

For a better digital health competence assessment, this scoping review focuses exclusively on instruments that assess multiple dimensions of digital health competencies, excluding only single-dimension measures (e.g., digital literacy) that capture a narrow slice of competence. This highlights which competencies are being assessed, the healthcare professionals targeted, and the contexts in which they are applied. Additionally, the use of rigorously developed and validated instruments is a cornerstone of high-quality measurement in health (21). This is essential to obtain reliable estimates of competence, evaluate and support educational interventions, and enable meaningful comparison across settings and professions. By examining how these instruments were developed and the psychometric properties reported (e.g., validity, reliability), this review provides a qualitative appraisal of each instrument that helps to identify conceptual and methodological gaps and to indicate priorities for future refinement, adaptation, and development. Strengthening the digital health competencies of healthcare professionals through robust assessment is therefore key not only to improving patient care but also to enabling effective, equitable, and sustainable public health strategies in the digital era (22).

This scoping review seeks to answer: “Which instruments have been developed and validated to assess digital health competencies among healthcare professionals?.” The aim of this scoping review is (i) to identify and map the instruments developed to assess the digital health competencies of healthcare professionals working in various healthcare settings, (ii) to analyse these instruments regarding the areas of digital health to which they apply, the development process and psychometric properties, and the competencies assessed, and (iii) to identify psychometrically validated instruments that adequately captures the breadth concept of digital health competencies.

## 2 Methods

### 2.1 Study concept

This scoping review has followed the JBI Manual for Evidence Synthesis (23). The eligibility criteria were defined by the Population, Concept and Context framework.

#### 2.1.1 Population

The population comprised healthcare personnel working across care settings. Eligible studies included those involving physicians, nurses, pharmacists, nutritionists, allied health professionals, and other healthcare staff engaged in direct patient care, as well as mixed-sample studies in which at least some participants were healthcare professionals involved in patient care. Studies exclusively involving health personnel who did not provide direct patient care (e.g., administration, finance, education) were excluded.

#### 2.1.2 Concept

The concept of interest in this scoping review is instruments that measure digital health competencies among healthcare professionals within digital health areas (e.g., eHealth, mHealth, telehealth, electronic health records) using the broad, multidimensional understanding defined earlier of digital competence in healthcare. Eligible studies developed, validated, or applied an instrument that assessed this multidimensional construct in healthcare professionals and reported two or more dimensions of competence under evaluation (e.g., knowledge, skills, attitudes). Studies were excluded if they did not report the development, validation, or use of an instrument specifically for healthcare professionals or if the instrument reported the assessment of only one dimension of competence.

#### 2.1.3 Context

The context for this scoping review includes clinical settings or mixed settings that combine clinical and non-clinical environments. Studies conducted exclusively in non-clinical environments (e.g., administration, finance, or purely academic/educational settings with no clinical component) or outside healthcare were excluded.

### 2.2 Search strategy

A comprehensive literature search was conducted in the following electronic databases: PubMed, Scopus, and Web of Science, using the key terms (Digital Health OR Digital Technology OR Telemedicine OR telehealth OR eHealth OR virtual medicine OR mHealth OR mobile health OR telecare OR tele-care OR tele care OR tele intensive care OR Distance Counselling OR Remote Consultation) AND (Professional Competence) AND (Health Personnel OR Nurses OR Physicians OR Caregivers OR Dental Staff OR Nutritionists OR Pharmacists OR Physical Therapists OR Psychotherapists OR Medical Laboratory Personnel OR healthcare worker OR healthcare workers). To consult the complete search, see the Supplementary Table S1. The databases were selected based on institutional accessibility, topic relevance, and comprehensive coverage of peer-reviewed literature. The search strategy was formulated in collaboration with a research librarian from the NOVA National School of Public Health, NOVA University Lisbon. The search included papers published within the last 10 years (2015–2025). This timeframe was selected to provide a clearer view of the instruments available to assess digital health competencies over the last 10 years, coinciding with the consolidation of major digital health competency frameworks published between 2016 and 2019 (3), and aligning with the period covered by the WHO Global Strategy on Digital Health (6), reflecting the growing interest in digital health competencies over the last decade.

Following the initial search, which established the primary set of studies, a supplementary search was conducted to capture additional relevant articles that complemented and refined the initial evidence base (Supplementary Table S1).

The inclusion criteria were studies that (i) developed, validated, or applied an instrument to assess healthcare professionals’ digital health competencies; (ii) were targeted at healthcare professionals; (iii) were conducted in various healthcare settings; (iv) were conducted in different geographical regions or countries; (v) were published in peer-reviewed journals and (vi) were published between 1st January 2015 and 26th June 2025. The exclusion criteria were studies (i) not published in English or Portuguese; (ii) commentaries, editorials, and opinion pieces with no empirical data; (iii) systematic reviews, meta-analyses, scoping reviews or narrative reviews; (iv) studies published before 1st January 2015.

### 2.3 Study/source of evidence selection

Following the literature search, all identified articles were uploaded to Rayyan software (24). This software was used throughout the whole selection process. After all duplicates were removed, the screening process was conducted in two phases. Phase one involved screening titles and abstracts to evaluate the relevance of the studies according to the eligible criteria, by two independent reviewers. Records with titles suggestive of eligibility but without an abstract were carried forward to full-text screening. A third reviewer resolved disagreements between the initial reviewers regarding the selected articles. Phase two involved a comprehensive full-text review of the studies identified in Phase one.

### 2.4 Data extraction

Data was extracted using a predefined data extraction sheet constructed for the purpose of this study. Data obtained from the full-text articles were organized according to the author(s) ID (author’s last name and year of publication), study design, country of origin of the study, healthcare professionals involved, and the healthcare setting. This data extraction and analysis was performed using Microsoft Excel. Additionally, data extracted for the results section was organized by instrument, population and sample size, setting, digital health area, dimensions of digital health competencies assessed, number of items, development method (refers to whether the instrument was previously developed (i.e., adopted or adapted), or developed in the included study), and psychometric properties reported. A column reflecting the inclusion for additional analysis was added.

Instruments were taken forward for analysis if (i) the complete questionnaire was available and (ii) the publication provided enough information to follow the Handbook of Recommended Practices for Questionnaire Development and Testing in the European Statistical System and the COSMIN guideline for systematic reviews of PROMs. Instrument development was analysed according to the Handbook’s five stages of questionnaire design and testing: conceptualization, questionnaire design, questionnaire testing, revision, and data collection (25), and the psychometric properties were mapped following the COSMIN nine measurement properties: content validity, structural validity, internal consistency, cross-cultural validity/measurement invariance, reliability, measurement error, criterion validity, hypotheses testing for construct validity, and responsiveness (26). Where applicable to each study, the two guidelines were used as reference frameworks to organize what was reported about development and psychometric properties. The guidelines served only for mapping, not for judging adequacy; evidence was presented descriptively, and no COSMIN risk-of-bias ratings or grading were performed.

## 3 Results

**Figure 1:**
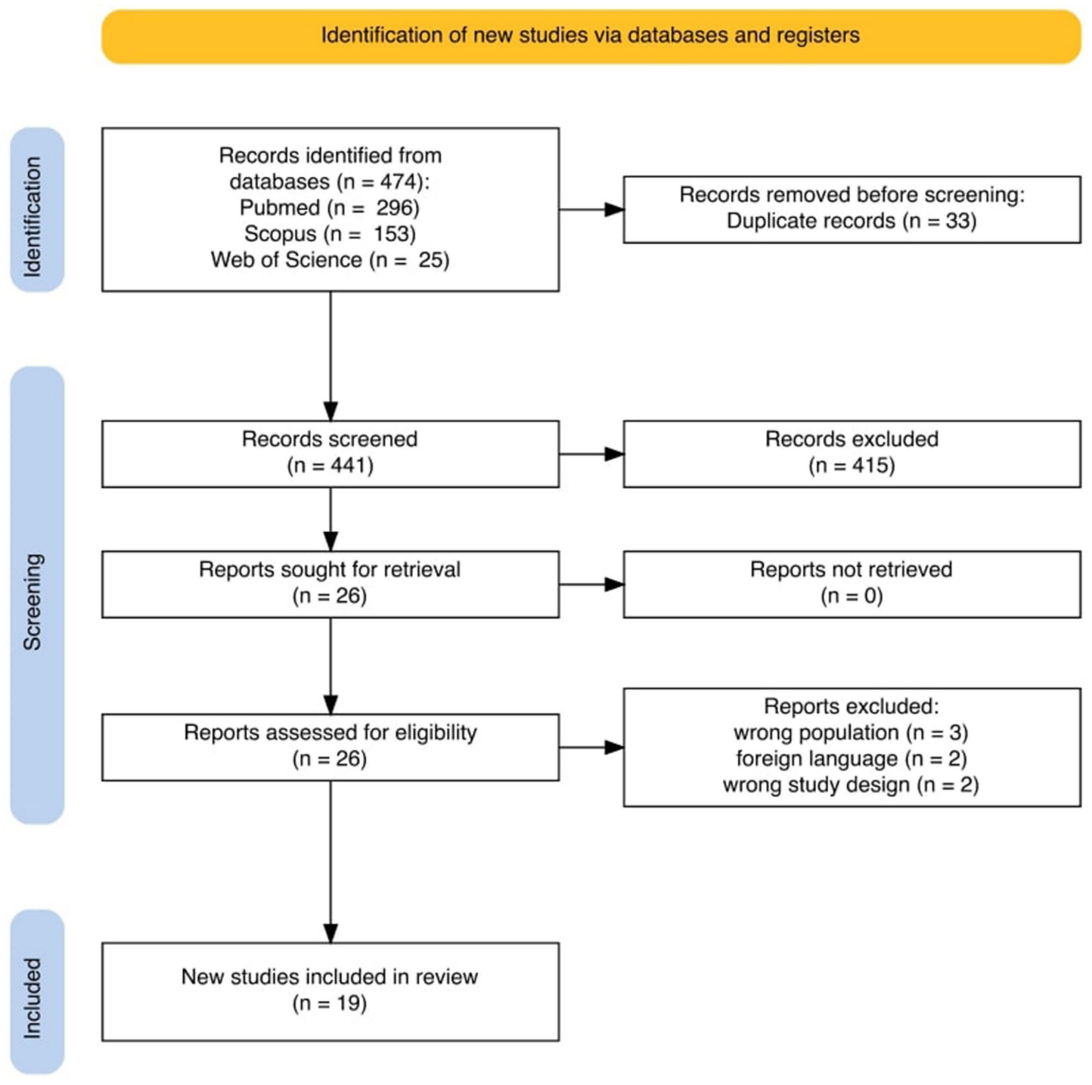
Prisma flowchart.

A total of 474 records were identified through database searching. After removal of 33 duplicates, 441 records were screened, of which 19 studies met the eligibility criteria (Figure 1).

### 3.1 Study characteristics

Most of the studies (14) were cross-sectional studies in design (27–40). Additionally, the other studies employed a range of other designs: one was an exploratory observational study (41) and two mixed-methods studies (42, 43).

Between 2018 and 2022, publication activity was modest with three studies published in 2018 (39, 40, 43); two studies published in 2019 (27, 28) and 2021 (30, 31); and one study published in 2020 (29) and 2022 (41). From 2023 onwards, the pace increased, with five studies in 2023 (33, 34, 36, 42, 44), two in 2024 (32, 45), and three in the first half of 2025 (35, 37, 38).

Regarding the geographical distribution, two studies were conducted in Canada (39, 43), three in Africa [two in Ethiopia (29, 34) and one in Libya (31)], two in Asia [one in Indonesia (42) and one in China (38)], nine in Europe [six in Finland (28, 30, 32, 33, 36, 37), one in the United Kingdom (35), one in the Netherlands (27), one in Spain (41)]; and three were conducted internationally (40, 44, 45).

Most of the studies (11) were performed in mixed settings, including hospitals, primary healthcare centres, social care, and others (28, 30, 32, 33, 35–39, 41, 43). Four studies were conducted exclusively in hospitals (27, 31, 34, 42) and one in public health centres (29). Three studies did not specify the setting (40, 44, 45). Most of the studies (9) focused on nurses (27, 28, 30, 36, 39, 42–45), followed by eight studies that addressed a combination of healthcare professionals, including nurses, physicians, pharmacists, physiotherapists and midwives, among others (29, 32–35, 37, 40, 41). One study focused on physicians (31) and one on paediatric healthcare workers and students (38). Eleven studies focused exclusively on professionals delivering direct patient care (27, 28, 30, 31, 35, 36, 39, 42–45), whereas eight involved professionals in both direct and non-direct care roles (29, 32–34, 37, 38, 40, 41). An overview of studies’ characteristics is provided in Table 1.

**Table 1:** Table of studies’ characteristics.

Author ID	Article name	Study design	Country	Year	“Population”	“Setting”
Thye et al., 2018 (40)	What Are Inter-Professional eHealth Competencies?	Cross-sectional study	International (Europe, Asia, North America, Central/South America Africa, Australia)	2018	Physicians; Nurses; Pharmacists; Other health care professionals or staff, Health executives; Health IT specialists; Leaders and experts from science and education	International online survey
Kleib and Nagle, 2018 (43)	Development of the Canadian Nurse Informatics Competency Assessment Scale and Evaluation of Alberta’s Registered Nurses’ Self-perceived Informatics Competencies	Exploratory, descriptive, cross-sectional study	Canada	2018	Nurses (registered nurses and registered psychiatric nurses)	Acute Care; Community; Other settings
Kleib and Nagle, 2018 (39)	Psychometric Properties of the Canadian Nurse Informatics Competency Assessment Scale (C-NICAS)	Cross-sectional study	Canada	2018	Nurses (registered nurses and registered psychiatric nurses)	Acute Care; Community; Other settings
van Houwelingen et al., 2019 (27)	Hospital Nurses’ Self-Reported Confidence in Their Telehealth Competencies	Cross-sectional study	Netherlands	2019	Nurses (registered nurses)	Hospital
Vehko et al., 2019 (28)	Experienced time pressure and stress: electronic health records usability and information technology competence play a role	Cross-sectional study	Finland	2019	Nurses (registered nurses)	Hospital; Primary care; Private practice; Social care; Other healthcare setting
Shiferaw et al., 2020 (29)	Healthcare providers’ digital competency: a cross-sectional survey in a low-income country setting	Cross-sectional study	Ethiopia	2020	Nurses; Health Officers; Medical laboratory professionals; Midwives; Pharmacists	Public Health Centres
Kaihlanen et al., 2021 (30)	Nursing informatics competences of Finnish registered nurses after national educational initiatives: A cross-sectional study	Cross-sectional study	Finland	2021	Nurses (registered nurses)	Emergency care; Psychiatric and substance abuse services; Specialised health care; Elderly care; Outpatients department; Other
Elhadi et al., 2021 (31)	Telemedicine Awareness, Knowledge, Attitude, and Skills of Health Care Workers in a Low-Resource Country During the COVID-19 Pandemic: Cross-sectional Study	Cross-sectional study	Libya	2021	Physicians	Hospital
Reixach et al., 2022 (41)	Measuring the Digital Skills of Catalan Health Care Professionals as a Key Step Toward a Strategic Training Plan: Digital Competence Test Validation Study	Exploratory observational study	Spain	2022	Dentists, dental hygienists and technicians; Dietitians-nutritionists; Occupational therapists; Nurses; Opticians or optometrist; Pharmacists; Physicians; Physiotherapists; Podiatrists; Speech therapists, and other health or clinical specialists (such as Biologists; Physicists or Chemists; and Psychologists)	Public and private healthcare providers
Jarva et al., 2023 (33)	Healthcare professionals’ digital health competence and its core factors; development and psychometric testing of two instruments	Cross-sectional study	Finland	2023	Registered nurse; Practical nurse; Physical therapist; Midwife; Occupational therapist; Public health nurse; Laboratory nurse/bio-analyst; Radiographer; Social worker; Paramedic; Social advisor; Other (rehabilitation advisor, podiatrist, prosthetist/orthotist); Assistant head nurse; Head nurse; Service manager/supervisor; Specialist/project coordinator	Inpatient (including hospital ward, emergency ward, intensive care unit, operating theatre, delivery room); Outpatient; Home care and assisted living; Emergency care; Administration and research; Other
Purba et al., 2023 (42)	Nurses perceived knowledge, self-confidence, and attitudes in using telemedicine: A case study from West Indonesia	Descriptive-quantitative cross-sectional study	Indonesia	2023	Nurses	Private Hospital
Mengestie et al., 2023 (34)	Health Information Technologies in a Resource-Limited Setting: Knowledge, Attitude, and Practice of Health Professionals	Cross-sectional study	Ethiopia	2023	Doctors; Nurses; Midwives; Laboratory Technicians; Pharmacists; Others	Hospital
Kinnunen et al., 2023 (36)	Nurses’ Informatics Competency Assessment of Health Information System Usage: A Cross-sectional Survey	Cross-sectional study	Finland	2023	Nurses (registered nurses, midwives, and public health nurses)	Inpatient ward; Outpatient care and virtual hospital; Emergency room and emergency care; Examination, operation, and labour; Mobile care and home healthcare
Golz et al., 2023 (44)	Content Validation of a Questionnaire to Measure Digital Competence of Nurses in Clinical Practice	Delphi study	International (Switzerland, Germany, Netherlands, United Kingdom, Austria, Italy)	2023	Nurses	Not specified
Golz et al., 2024 (45)	Psychometric Validation of the Digital Competence Questionnaire for Nurses	Cros-sectional study	International (United States of America, United Kingdom, Australia, Switzerland, Canada, Ghana, Indonesia, and other countries)	2024	Nurses	Not specified
Jarva et al., 2024 (32)	Healthcare professionals’ digital health competence profiles and associated factors: A cross-sectional study	Cross-sectional study	Finland	2024	Registered nurse; Practical nurse; Physical therapist; Midwife; Occupational therapist; Public health nurse; Laboratory nurse/bio-analyst; Radiographer; Social worker; Paramedic; Social advisor; Other (rehabilitation advisor, podiatrist, prosthetist/orthotist); Assistant head nurse; Head nurse; Service manager/supervisor; Specialist/project coordinator	Inpatient (including hospital ward, emergency ward, intensive care unit, operating theatre, delivery room); Outpatient; Home care and assisted living; Emergency care; Administration and research; Other
Ylönen, et al., 2025 (37)	Social services and healthcare personnel’s digital competence profiles: A Finnish cross-sectional study	Cross-sectional study	Finland	2024	Nurse (registered, assistant or specialist); Social worker, sociologic, curator, school psychologist; Medical doctor or dentist; Rescue worker (fireman, etc.); Support service worker (secretary, developer, etc.); Other profession	Social services and healthcare organisation’s
Ren et al., 2025 (38)	Digital competency among pediatric healthcare workers and students: a questionnaire survey	Cross-sectional study	China	2025	Paediatric healthcare workers and students	Tertiary children’s hospital; Medicine College
Erfani et al., 2025 (35)	Factors influencing digital health competence among healthcare professionals: A cross-sectional study	Cross-sectional study	United Kingdom	2025	Nurse; Midwife; Physiotherapist; Occupational Therapist; Other	Hospital, Non-hospital, Other

### 3.2 Instruments assessing digital health competencies identified

In total, 14 instruments assessing healthcare professionals’ digital health competencies were identified. Some instruments are documented in paired publications, with the development and psychometric testing presented separately. Eight instruments did not focus on a specific area; they assessed general digital health competencies rather than anchoring measurement to a single area (29, 32, 33, 35, 37, 38, 41, 44, 45), three focused on telemedicine/telehealth (27, 31, 42), six on health informatics (28, 30, 34, 36, 39, 43), and one on eHealth (40). For synthesis purposes, studies were classified as “general” when the instrument did not target a specific digital health area, and as “health informatics” when the instrument assessed informatics-related competencies within healthcare practice. An overview of the instruments identified is summarized in Table 2.

**Table 2:** Results table.

Instrument	Purpose	Population and sample size	Setting	Digital health area	Dimensions of digital health competencies assessed	Number of items	Development method	Psychometric properties reported	Included for analysis
Telemedicine - Awareness, Knowledge, Attitude and Skills (AKAS) Questionnaire (31)	Provide an overview of physicians’ awareness, knowledge, attitude, and skill in using telehealth services in Libya	Physicians (*N* = 673)	Hospital	Telemedicine	Awareness; Knowledge; Attitude; Skills	47	Previously developed	Internal consistency	Included
EU*US eHealth Work survey (40)	Investigate which competencies are at the intersection of the individual groups of health professionals	Healthcare professionals (*N* = 718)	Not specified	eHealth	Applications in patient care; IT background knowledge of health professionals; Education and research; management; Interpersonal and social dimensions	33*	Previously developed	Not reported	Not included
Canadian Nurse Informatics Competency Assessment Scale (C-NICAS) (39, 43)	Develop a research-based informatics assessment scale based on the CASN entry-to-practice competencies, and to apply this scale to assess self-perceived informatics competencies among RNs in Alberta	Nurses (*n* = 2,844)	Various	Health informatics	Foundational ICT skills; Information and knowledge management; Professional and regulatory accountability; Use of ICT in delivery of patient care	21	Developed	Content validity; Structural validity; Internal consistency	Included
Telehealth KSA (knowledge, skills and attitudes) (27)	Describe how hospital nurses self-rate their confidence in essential telehealth KSAs, summarized as telehealth care competence	Nurses (*N* = 1,017)	Hospital	Telehealth	Knowledge; Skills; Attitudes	31	Developed	Content validity	Included
Nurses’ Information System Survey 2017^†^ (28)	Explore the associations of EHR usability factors and nurses’ informatics competence factors with self-reported time pressure and psychological distress among registered nurses	Nurses (*N* = 3,607)	Various	Health informatics	Classification competence; e-care competence; e-documentation competence; e-ethics competence	16	Previously developed and adapted	Internal consistency	Not included
Questionnaire adopted from European commission’s digital competency framework^‡^ (29)	Assess digital competency of healthcare providers among seven public health centers in North-West Ethiopia	Healthcare professionals (*N* = 167)	Public Health centers	General	Information and data literacy; Communication and collaboration; Digital content creation; Safety; Problem-solving	22	Developed	Content validity; Internal consistency	Not included
Nursing informatics competences questionnaire (30)	Examine whether nurses who graduated after the Finnish educational initiatives have higher nursing informatics competences than nurses who graduated before the initiatives	Nurses (*N* = 1,639)	Various	Health informatics	Terminology-based documentation; Patient-related digital work; General IT; Electronic documentation	4	Developed	Internal consistency	Included
Digital Competence Test (41)	Validate the digital competence test developed *ad hoc* and measure the digital competence level of Catalan HPs to establish their current level as the baseline for designing a strategic training plan	Healthcare Professionals (*N* = 803)	Various	General	Search, select and contrast information with digital tools; Organise the information and data with digital tools; Analyse, exploit and visualize data with digital tools; Interact and share information and digital content; Collaborate with others using digital technologies; Participate in the social and cultural transformation through the digital society; Create and edit digital content; Design, integrate and rework digital content with various formats; Design and manufacture objects through digital technology; Protect systems, devices and digital content; Protect personal data and privacy; Act civically in the digital environment; Understands the basics and uses digital technology; Identifies personal and professional needs and applies digital solutions	Case 1: 7; Case 2: 11	Developed	Content validity	Included
DigiHealthCom and DigiComInf (33)	Develop and psychometrically validate two self-assessed instruments measuring digital health competence and factors associating with it	Healthcare Professionals (*N* = 643)	Various	General	DigiHealthCom: Human-centred remote counselling competence; Digital solutions as part of work; Information and communication technology (ICT) competence; Competence in utilizing and evaluating digital solutions; Ethical competence related to digital solutions. DigiComInf: Support from management; Organisational practices as part of digital competence development; Colleagues’ adoption and influence	DigiHealthCom: 42, DigiComInf: 15	Developed	Content validity; Structural validity; Internal consistency	Included
TeleOSCE questionnaire (42)	Determine the knowledge, self-confidence, and attitudes in using telemedicine according to nurses’ perceptions in a private hospital in Indonesia	Nurses (*N* = 52)	Hospital	Telemedicine	Knowledge; Self-confidence; Attitude	22	Previously developed	Internal consistency	Included
Questionnaire to measure knowledge, attitude, and practice of health information technology^‡^ (34)	Determine the knowledge, attitude, and practice of health information technology among health professionals	Healthcare Professionals (*N* = 347)	Hospital	Health informatics	Knowledge; Attitude; Practice of health information technology	38	Developed	Content validity; Structural validity; Internal consistency	Not included
Nurse survey on information systems 2020 (36)	Describe nurses’ perceptions of their informatics competencies regarding health information system usage	Nurses (*N* = 610)	Various	Health informatics	Nursing documentation; Digital environment; Ethics and data protection	16	Previously developed and adapted	Content validity; Structural validity; Internal consistency	Included
Digital Competence Questionnaire (DCQ) (44, 45)	Identify items for an item pool of a questionnaire to measure clinical practice nurses’ digital competence and to evaluate the content validity, and evaluate the structural validity and internal consistency of the Digital Competence Questionnaire (DCQ) for Clinical Practice Nurses	Nurses (*N* = 185)	Not specified	General	Knowledge; Skills; Attitudes	12	Developed	Content validity; Structural validity; Internal consistency	Included
Paediatric students and paediatricians attitude towards digital competency (38)	Investigate digital perceptions and competencies among medical students in pediatrics and pediatric healthcare workers in China	Healthcare professionals and students (*N* = 518)	Hospital, Medicine College	General	Digital awareness; Digital capability; Digital application; Digital content innovation; Ethic issues related with digital technology use	49	Developed	Content validity; Internal consistency	Included

*Only the number of items in the competence module applied in the study. † Instrument in Swedish and Finnish. ‡ Instrument not available.

Five studies reported the development and psychometric validation of instruments: the Canadian Nurse Informatics Competency Assessment Scale (C-NICAS) (39, 43), the Digital Health Competence (DigiHealthCom) and Factors Associated with Digital Health Competence (DigiComInf) instruments (33), and the Digital Competence Questionnaire (DCQ) (44, 45).

Three studies reported the use of the DigiHealthCom and DigiComInf instruments. Erfani et al. (35) used culturally and linguistically adapted British English versions of these instruments and conducted additional validation; Jarva et al. (32) and Ylönen et al. (37) relied on the versions previously developed and validated by the authors’ earlier work and did not report any new validation.

Two studies reported the development of new instruments, the Paediatric students and paediatricians’ attitude towards digital competency questionnaire (38), which established content validity and assessed reliability, and the Telehealth Knowledge, Skills and Attitudes Questionnaire (KSA) (27), which reported only on clarity and relevance via expert interviews. One study reported the development of a questionnaire to measure knowledge, attitude, and practice of health information technology, reporting content validity, structural validity and internal consistency (34).

One study adapted a national physician survey to nurses and added a competence module, reporting the internal consistency for the module’s four dimensions; this version was administered in Swedish/Finnish (28). Another study included in this review reported the use of an updated version of this module in English, reporting the internal consistency (36).

One study developed a questionnaire adapted from the European Commission’s DigComp framework (29) and another study developed a questionnaire with competencies identified by a previous study (30); both reported internal consistency for the overall scale only. One study developed a digital competence test, and the internal consistency reported was based only on the greatest lower bound given the lack of homogeneity of the scoring scale (41).

Three studies used previously developed instruments: the Telemedicine Awareness, Knowledge, Attitude, Skills (AKAS) Questionnaire (31), reporting reliability; the EU*US eHealth Work survey (40), for which only the competencies section was administered, and no psychometric properties were reported; and the Telemedicine Objective Structured Clinical Exam (TeleOSCE) questionnaire, translated into Indonesian, with internal consistency reported (42).

### 3.3 Instrument development and measurement properties reported

Of the 14 instruments identified, 10 met the inclusion criteria and were retained for analysis (Table 2). The following analysis will be reported by the digital health area that the instruments included are designed to assess. Of the 10 instruments included for analysis, three measured competencies related to telemedicine (27, 31, 42), three competencies related to health informatics (30, 36, 39, 43), and four competencies without a specific area attached, “General” (33, 38, 44, 45). The reporting of the nine measurement properties defined in the COSMIN guidelines for each included study is summarized in Table 3.

**Table 3:** COSMIN measurement properties reported in the included studies.

COSMIN instrument	Content validity	Structural validity	Internal consistency	
			Per dimension	Total scale
Telemedicine—Awareness, Knowledge, Attitude & Skills (AKAS) Questionnaire (31)	Not reported	Not reported	Awareness: *α* = 0.823; Knowledge: *α* = 0.735; Attitude: *α* = 0.910; Skills: α = 0.950	Not reported
TeleOSCE questionnaire (42)	Not reported	Not reported	Knowledge: α = 0.841; Self-confidence: α = 0.899; Attitude: α = 0.774	Not reported
Telehealth KSA (knowledge, skills and attitudes) (27)	Expert interviews (*N* = 3). Cognitive interviews	Not reported	Not reported	
Canadian Nurse Informatics Competency Assessment Scale (C-NICAS) (39, 43)	Expert consensus following literature review and stakeholder consultation. Pre-test (*N* = 12)	KMO = 0.94. PCA: four-factor structure, eigenvalues greater than 1 and explained 61.04% of the variance	Foundational ICT skills: α = 0.81; Information and knowledge management: α = 0.85; Professional and regulatory accountability: α = 0.81; Use of ICT in delivery of patient care: α = 0.87	α = 0.93
Nurse survey on information systems (36)	Pilot test (*N* = 10)	EFA: three-factor structure	Nursing documentation: α = 0.93; Digital Environment: α = 0.86; Ethics and data protection: α = 0.82	
Nursing informatics competences questionnaire (30)	Not reported	Not reported	Not reported	α = 0.73
DigiHealthCom (33)	Expert panel (*N* = 17). Pilot study (*N* = 20). I-CVI varied between 0.77 to 1 when measuring relevance; and 0.71 to 1 when measuring clarity. S-CVI/Ave of 0.94 for relevance and 0.96 for clarity	KMO = 0.96. EFA: five-factor model, eigenvalues greater than 1 and explained 68.9% of the variance	Human-centered remote counselling competence: α = 0.97, Digital solutions as part of work: α = 0.91, Information and communication technology (ICT) competence: α = 0.93, Competence in utilizing and evaluating digital solutions: α = 0.92, Ethical competence related to digital solutions: α = 0.91	α = 0.95
DigiComInf (33)	Expert panel (*N* = 17). Pilot study (*N* = 20). I-CVI varied between 0.75 to 1 when measuring relevance; and 0.86 to 1 when measuring clarity. S-CVI/Ave of 0.95 for relevance and 0.96 for clarity	KMO = 0.89. EFA: three-factor model, eigenvalues greater than 1 and explained 59.6% of the variance	Support from management: α = 0.88, Organisational practices as part of digital competence development: α = 0.79, Colleagues’ adoption and influence: α = 0.74	α = 0.94
Digital Competence Questionnaire (44, 45)	Literature review and Delphi study (*N* = 27). I-CVI varied between 0.81 to 1. S-CVI/Ave = 0.95	KMO = 0.83. EFA: two-factor model. Attitude explained 33% of the variance, Knowledge & Skills explained 24%	Attitude: α = 0.91; Knowledge & Skills: α = 0.81	Not reported
Paediatric students and paediatricians attitude towards digital competency (38)	Literature review and Delphi expert survey. Pre-test (*N* = 60)	KMO = 0.710	Not reported	α = 0.973
Digital Competence Test (41)	Interactive expert consultation, and pilot tests. Cross-validation (*N* = 8)	Not reported	Not reported*	

α Cronbach’s alpha; KMO Kaiser–Meyer–Olkin; PCA Principal component analysis; EFA Exploratory factor analysis; I-CVI Item-level content validity index; S-CVI Scale-level content validity index. *The internal consistency of the survey was only reported by the greatest lower bound, given the lack of homogeneity of the scoring scale, and this coefficient is not explicitly recognised in the COSMIN guidelines as a measure of internal consistency. †The other measurement properties, including Cross-cultural validity/measurement invariance, Reliability, Measurement error, Criterion validity, Hypotheses testing for construct validity, and Responsiveness were not identified in the available reports for the included instruments.

#### 3.3.1 Telemedicine

The AKAS questionnaire (31) has 47 items and was applied in a sample of hospital physicians, and methodological details of the adaptation process for a healthcare context were not described, as the instrument was initially developed for use in teaching hospitals (46). A pre-test to assess item comprehension and clarity, as well as a subsequent revision phase based on such feedback, were not reported. Data was collected electronically, and usability or functionality testing was not mentioned. The study reported evidence for internal consistency, with Cronbach’s alpha values of 0.823 for Awareness, 0.735 for Knowledge, 0.910 for Attitude, and 0.950 for Skills. Content validity, test–retest reliability, measurement error, hypotheses testing for construct validity, cross-cultural validity/measurement invariance, criterion validity, and responsiveness were not provided. The competencies assessed are related to the level of awareness regarding the context and basic concepts of telemedicine (e.g., “Telemedicine provides health care services where distance is a problem”), the knowledge of what telemedicine is and its clinical applications (e.g., “Telemedicine is the use of telecommunication to provide medical information and services”), the attitude related to how telemedicine affects care delivery and the health system, and perceived professional benefits (e.g., “Use of telemedicine could make the distribution of healthcare more even with more emphasis on prevention”), and the operational technology skills needed to run telemedicine (e.g., “Establish connectivity”), with different scoring per section.

The TeleOSCE (42) is a 22-item questionnaire adapted and translated, but the methodological details of the translation process were not described. A pre-test was not reported, and while data collection was electronic, specific usability or functionality testing was not mentioned. Although the authors stated that the questionnaire was valid and reliable, the study only reported evidence for internal consistency, with Cronbach’s alpha values of 0.841 for Knowledge, 0.899 for Self-confidence, and 0.774 for Attitude. Evidence for content validity, test–retest reliability, measurement error, hypotheses testing for construct validity, cross-cultural validity/measurement invariance, criterion validity, and responsiveness was not provided. The TeleOSCE assesses knowledge of legal and operational requirements for the use of telemedicine (e.g., “I have a full understanding of federal laws and regulations concerning informed consent for telemedicine”), the self-confidence to carry out telemedicine tasks (e.g., “I feel very confident in communicating effectively with a patient via telemedicine”), and the attitude towards the perceived value of telemedicine (e.g., “I think telemedicine is a good alternative to face-to-face healthcare”), scoring from strongly agree to strongly disagree.

The Telehealth KSA (27) is a 31-item questionnaire developed to self-rate confidence in telehealth knowledge, skills, and attitudes. Content validity was addressed qualitatively before data collection through expert interviews with three nurse specialists and cognitive interviews with hospital nurses leading to item refinement and reduction from an earlier list. The instrument was administered electronically without reported usability or functionality testing. Structural validity, internal consistency, test–retest reliability, hypotheses testing for construct validity, cross-cultural validity/measurement invariance, measurement error, criterion validity, and responsiveness were not reported. The instrument covers the knowledge of core legal/operational basics and how telehealth fits into care pathways (e.g., I have knowledge of policies, procedures, and protocols of the organization concerning the deployment of telehealth technologies”), skills for practical delivery and patient interaction online (e.g., “I am able to use electronic health records”), and attitudes regarding innovation, ethical conduct, and self-confidence (e.g., “I am open minded about using new innovations in IT”) The competencies were rated from totally disagree to totally agree.

#### 3.3.2 Health informatics

The C-NICAS (39, 43) is a 21-item instrument developed based on the national entry-to-practice informatics competencies from the Canadian Association of Schools of Nursing (CASN). A pre-test for face validity was conducted with 12 nurses, and their feedback led to several modifications, including the addition of a ‘Not Applicable’ option and adjustments to the rating scale and item wording. However, specific usability or functionality testing of the electronic data capture was not reported. Strong evidence for structural validity was reported with Kaiser–Meyer–Olkin (KMO) values (0.94) and through an exploratory principal component analysis, which identified a clear four-factor structure explaining 61.04% of the variance. Evidence for internal consistency was also strong, with Cronbach’s alpha values of 0.93 for the total scale and 0.81 for Professional and regulatory accountability and Foundational ICT skills, 0.85 for Information and knowledge management and 0.87 for Use of ICT in delivery of patient care. Additionally, the study did not provide evidence for test–retest reliability, measurement error, hypotheses testing for construct validity, cross-cultural validity/measurement invariance, criterion validity, and responsiveness. The C-NICAS assessed competencies related to the use of ICT devices/applications (e.g., Computers/emails), the use of these technologies in line with professional standards, legal requirements, and workplace policies (e.g., “Comply with legal and regulatory requirements, ethical standards, and organizational policies”) and in providing patient care (e.g., “Use ICTs in a manner that supports the nurse–patient relationship”), and leveraging relevant information and knowledge to deliver informed patient care (e.g., “Describes the processes of data gathering, recording and retrieval in paper and electronic records”), scoring from not competent to very competent.

The nurse survey on information systems (36) is a 16-item adapted and updated version of a previous questionnaire. A pilot test was conducted with 10 nurses to assess the clarity of the items, and revisions were made based on their feedback. Usability or functionality testing of the electronic platform was not mentioned. Structural validity was reported through an exploratory factor analysis, which identified a clear three-factor structure. Evidence for internal consistency was also robust, with Cronbach’s alpha values of 0.93 for Nursing documentation, 0.86 for Digital environment, and 0.82 for Ethics and data protection. Evidence for test–retest reliability, measurement error, hypotheses testing for construct validity, cross-cultural validity/measurement invariance, criterion validity, and responsiveness. Was not provided. This survey evaluates nurses’ self-assessed mastery of skills required by information systems and covers competencies related to documentation along the nursing care process according to national classifications (e.g., “Documentation of nursing diagnosis (FiCND)”); to work in digital settings (e.g., “Basic IT skills); and to ethics and data protection/security (e.g., “Compliance with data protection and data security principles in daily work”), scoring from weak to excellent.

The nursing informatics competences questionnaire (30) has a brief 4-item self-report to assess nurses’ informatics competence. A pre-test was not conducted; and data collection was made electronically, with usability or functionality testing not reported. Internal consistency was reported only for the overall score, with a Cronbach’s alpha of 0.73 for the scale. Content validity, structural validity, test–retest reliability, measurement error, hypotheses testing for construct validity, cross-cultural validity/measurement invariance, criterion validity, and responsiveness. Were not reported. The survey assesses competencies regarding how well nurses master documentation; support patients with electronic services; and basic IT skills, scoring from very poorly to very well.

#### 3.3.3 General

The development of the DigiHealthCom and DigiComInf instruments (33) followed a rigorous three-phase process. The initial conceptualization was grounded in systematic reviews and qualitative interviews with healthcare professionals. This was followed by a comprehensive pre-testing phase that included a formal content validity assessment by a panel of 17 experts using the content validity index (CVI) method for the items (I-CVI) and the total scales (S-CVI), and a separate pilot study with 20 professionals to ensure face validity and comprehensibility. Feedback from both stages led to significant revisions and refinement of the items before the final instruments were established. The study reported strong evidence for structural validity, with KMO values and an exploratory factor analysis confirming a five-factor structure for the DigiHealthCom (explaining 68.9% of variance) and a three-factor structure for the DigiComInf (explaining 59.6% of variance). Internal consistency was also reported, with Cronbach’s alpha ranging from 0.91 to 0.97 for the DigiHealthCom factors, and 0.76 to 0.88 for the DigiComInf factors. The study did not provide evidence for test–retest reliability, measurement error, hypotheses testing for construct validity, cross-cultural validity/measurement invariance, criterion validity, and responsiveness. The DigiHealthCom instrument evaluates the interpersonal and clinical skills needed for effective online patient interaction (e.g., “I can act in reciprocal (aiming towards respect and equality) interaction with the customer in remote counselling”); the attitudes towards digital solutions (e.g., “I am motivated to use digital solutions in my work”); proficiency in fundamental IT skills (e.g., “I can use the most common computer programs and services (e.g., email, intranet) in my work”); the ability to utilise and evaluate digital solutions (e.g., “I can critically evaluate new digital solutions”); and an understanding and application of ethical considerations related to digital tools (e.g., “I can secure the customer’s privacy when using digital solutions”). The DigiComInf instrument assesses factors associated with professional development in digital areas (e.g., “My manager supports the implementation of digital solutions”); organizational practices that foster digital competence development (e.g., “Digital competence development is planned in my unit according to individual needs”); and the influence of colleagues on professional development (e.g., “Colleagues are not reluctant to start using digital solutions at work”), scoring from completely disagree to completely agree.

The Digital Competence Questionnaire (DCQ) (44, 45) was developed through a rigorous two-stage process. An initial item pool was generated based on a literature review and a conceptual framework including knowledge, skills and attitude. The instrument then underwent a comprehensive pre-testing phase to establish content validity, which involved a three-round Delphi study with an international panel of experts. This process resulted in a 26-item pool reporting the CVI for the scale (0.95) and for each item (I-CVI). No specific usability or functionality testing of the survey platform was reported. A subsequent study provided strong evidence for structural validity with an exploratory factor analysis conducted, leading to a refined 12-item instrument with a clear two-factor structure (“Knowledge & Skills” and “Attitude”) that explained 57% of the variance. The study also reported internal consistency, with Cronbach’s alpha values of 0.81 and 0.91 for the two factors, respectively. Evidence was not provided for test–retest reliability, measurement error, hypotheses testing for construct validity, cross-cultural validity/measurement invariance, criterion validity, and responsiveness. The DCQ assesses the self-perceived knowledge of and confidence in using digital technologies for clinical tasks (e.g., “I feel confident about using digital technology to obtain data and information on clinical care”), and the nurses beliefs about the value and benefits of digital technology in clinical practice (e.g., “I believe that digital technology provides numerous benefits in terms of quality of care”), scoring from not relevant to very relevant.

The paediatric questionnaire (38) is a 49-item and was newly designed for this study, with its development based on an extensive literature review and a Delphi expert survey, and it was subsequently pre-tested with a sample of 60 students. Usability or functionality testing of the online survey platform was not described. For internal consistency, an overall Cronbach’s alpha of 0.973 was reported for the total scale. However, the study did not report the internal consistency values for each of the five individual subscales. The authors confirmed the data was suitable for factor analysis during the pre-test but only reported the KMO value of 0.710. Additionally, the study did not provide evidence for test–retest reliability, hypotheses testing for construct validity, cross-cultural validity/measurement invariance, measurement error, criterion validity, and responsiveness. The digital competency questionnaire assesses digital awareness of the role of technology in the medical field (e.g., “I have a good perception of the importance of digital technology in medicine”), digital capability for practical use and collaboration (e.g., “I am able to use digital tools for collaborating with colleagues”), digital application of technologies in clinical practice (e.g., “I feel confident using digital tools like telehealth in patient care”), digital content creation (e.g., “I have the ability to create new digital content for my work”), and understanding of ethical issues (e.g., “I am familiar with the ethical guidelines for using digital technology with patients”), scoring from strongly agree to strongly disagree.

The Digital Competence Test (41) is an objective digital competence test developed based on the Accreditation of Competence in ICTs (ACTIC) certificate through interactive expert review and pilot tests. Data collection was web-based, and usability/functionality was not reported. The psychometric results were limited to internal consistency only via Greatest Lower Bound (GLB) with a score of 0.66, lacking Cronbach’s alpha values. Structural validity, internal consistency, test–retest reliability, hypotheses testing for construct validity, cross-cultural validity/measurement invariance, and measurement error, criterion validity, and responsiveness were not reported. The test is a digital skills-level test based on scenarios, evaluating abilities in searching, organizing and analysing data, collaborating digitally, creating content, protecting digital information, and understanding the use of digital technologies with a scoring scale from initial to advanced levels.

## 4 Discussion

The rapid digital transformation of healthcare systems worldwide has underscored the need for healthcare professionals to possess robust digital health competencies. These competencies are essential not only for the effective use of digital technologies in clinical practice but also for ensuring patient safety, data security, and ethical decision-making in increasingly digital environments (47). As such, the development and validation of instruments to assess these competencies have become a critical area of research.

This scoping review sought to explore the landscape of available instruments and the following discussion addresses each of the scoping review objectives in turn, highlighting key patterns, gaps, and implications for future research and practice.

### 4.1 Identification and mapping of instruments

Most studies were conducted in mixed healthcare settings, with hospitals consistently included, even when not the sole focus, suggesting that digitalization may have a greater impact or visibility in hospital environments. The research is dominated by mixed samples, suggesting that the assessment involves a broad range of healthcare profiles. Studies targeting single professional groups most frequently focused on nurses, likely due to their large representation in the global health workforce (48), which facilitates sampling and educational interventions. Additionally, nursing benefits from more targeted digital health competency frameworks, which may encourage program development and evaluation studies (3). The literature already consolidates 30 digital health competency frameworks and although most instruments were developed without explicitly referencing existing frameworks, the dimensions they assess closely align with the cross-framework domains synthesized by Nazeha et al. (3) including knowledge of informatics concepts and processes, attitudes/awareness, documentation, communication, ethics/legal/regulatory requirements, and privacy/security. This alignment suggests a shared underlying structure and provides an empirical bridge between disparate instruments and a generalizable digital health competency framework applicable across professions and care settings, while preserving comparability across instruments.

### 4.2 Analysis of application areas, development processes, and psychometric properties

The instruments reviewed were predominantly applied in hospital settings, with public health contexts notably underrepresented.

Only one study was conducted in public health centers, and among the mixed-setting studies none explicitly included public health settings, although two did include public health nurses within their samples. Notably, the study set in public health centers (29) adopted the European Commission’s DigComp framework for citizens (16) to assess digital health competencies, underscoring the absence of purpose-built frameworks for this field.

Overall, digital health competencies specific to public health remain underexplored (3). Another study found that few frameworks focus specifically on digital public health (DiPH) and emphasized that the public health workforce needs appropriate competencies to steer digitalization effectively, with ethics highlighted as a core dimension and competency frameworks recommended to be grounded in professionals’ attitudes, knowledge, and skills (49). As noted earlier, these dimensions are reflected in several of the instruments analysed, even though most were not specifically targeted at public health professionals, further reinforcing the need for a dedicated digital public health competency framework.

Moreover, many of these instruments were not design for specific areas such as telemedicine or health informatics. This trend suggests a shift away from specialized areas toward a more comprehensive set of digital competencies relevant to a wider range of healthcare professionals. Nazeha et al. (3) also noted that distinctions among competencies related to eHealth, telehealth, or health informatics may be superficial, as the definitions and terminologies often overlap across frameworks.

### 4.3 Identification of psychometrically validated and comprehensive instruments

Aligned with the objective of identifying psychometrically validated instruments that capture the breadth of digital health competencies, the review found that despite growing attention in this field, rigorously developed and validated instruments remain limited. Most of the studies focused on developing new instruments, with only a few providing a more detailed psychometric reporting (33, 39, 43–45). Psychometric properties reported were mostly limited to content validity usually based on literature reviews, expert consultations and CVI values; structural validity reported mostly with KMO values and EFA/PCA analysis, and internal consistency typically with Cronbach’s alpha values.

Among the more robust instruments, DigiHealthCom and DigiComInf (33) stand out for their applicability across diverse healthcare professionals, including those in public health. These instruments uniquely incorporate organizational and social determinants of digital health competence, such as management support, training opportunities, and peer influence, reflecting evidence that digital readiness depends not only on individual skills but also on enabling conditions and social context (33, 50). Additionally, these instruments were the only ones among those included that explicitly followed the COSMIN guidelines to guide the study process and enhance its methodological quality.

Adherence to established validation guidelines, such as those proposed by the COSMIN guidelines (26) and the ESS Handbook (25), can enhance the scientific rigor of instrument development, improve transparency for future users, and facilitate adaptation and re-validation across different contexts and professional groups. Recent questionnaire development efforts in related fields have also followed the COSMIN guidelines, underscoring the importance of systematically addressing all relevant psychometric properties to ensure reliability and applicability to new settings and populations (51).

### 4.4 Strengths

To our knowledge, this is the first consolidation and analysis of existing instruments designed to assess digital health competencies among healthcare professionals. This study draws a clear conceptual boundary by focusing on instruments that treat digital health competence as a multidimensional construct, thereby offering a more accurate reflection of the complex skill set required in contemporary healthcare practice.

Although the term still lacks a single, settled definition, the evidence consolidated here shows it is inherently multi-faceted, interlocking dimensions such as awareness, attitudes, ethics and data protection, knowledge, information and data literacy, support from management, and collaboration. By limiting inclusion to instruments that assess multiple dimensions, this review not only mirrors this complexity but also reinforces the need for comprehensive assessment approaches.

This review also provides a comprehensive mapping of which competencies are being assessed, which healthcare professionals are targeted, and in which settings. This yields a clear overview of current coverage and highlights critical gaps. By examining the development processes and reported psychometric properties of the instruments, the review offers a concise yet informative synthesis of their methodological robustness. This enables researchers and practitioners to make informed decisions when selecting or adapting instruments for specific assessment needs.

Applying established methodological frameworks, such as COSMIN and the ESS Handbook, as analytical lenses also allowed for the identification of missing steps in development and validation processes. This not only underscores areas for improvement in existing instruments but also provides practical guidance for future tool development and adaptation. Collectively, these contributions help organise fragmented evidence into a usable framework, advancing the field toward more standardized and practice-relevant assessments of digital health competencies.

### 4.5 Implications for workforce training, education, and future research

Importantly, these findings have direct implications for public health workforce education and preparedness for an increasingly digital environment. They offer actionable insights for curriculum design, continuing professional development, and organizational readiness, and ultimately to contribute to build a competency framework focused on DiPH. As Brînzac, et al. have noted, “the lack of a comprehensive and well-developed DiPH competency framework remains a critical gap” (49). This review represents a foundational step toward addressing that gap by mapping existing instruments and identifying their relevance to public health contexts.

Iyamu et al. (22) argue for establishing a common baseline of general digital competencies across the public health workforce, beyond digital literacy. This scoping review contributes by distinguishing digital literacy from broader digital competencies. It shifts the focus from basic literacy to a more nuanced understanding of competence, offering a broader foundation for future research and practice. Given that public health relies on multidisciplinary, multi-professional approaches to prevention and health promotion (52), and that DiPH represents the population-level of digital health, the instruments identified here, though not DiPH specific, can serve as valuable reference points for the development of tailored instruments. Many of the dimensions they assess (e.g., attitudes, skills, knowledge, ethics) are directly relevant to the competency needs of the public health workforce (53).

### 4.6 Limitations

Regarding the limitations, this scoping review was designed to map instruments and their reported evidence; it did not include a formal critical appraisal of methodological quality. As such, it provides a descriptive synthesis of the landscape rather than a definitive evaluation of instrument quality. For several instruments, publicly available documentation on development and psychometric testing was limited or absent, which hindered the verification of key aspects such as content validity, construct validity, and reliability.

Because the mapping relied on published literature, some relevant details may exist in unpublished or inaccessible sources; clarifying these would likely require direct correspondence with instrument authors or access to Supplementary materials. Consequently, this review does not make conclusive judgments about the quality of individual instruments but instead highlights reporting gaps and areas where further evidence is needed. Importantly, the absence of a reported step does not invalidate an instrument but indicates a reporting gap against the applied criteria and highlights where improvement or further evidence would be most valuable. In some cases, missing steps can suggest clear priorities for additional development.

Additionally, some psychometric tests and procedures described in primary studies may not have been reflected in our findings because they are not specified by the guidelines applied; thus, the choice of guideline shapes which evidence is extracted and how it is interpreted. Furthermore, the applicability of the psychometric properties described in the COSMIN guidelines may vary across instruments. In the results section, the label “not reported” for a given COSMIN measurement property indicates that no data consistent with the COSMIN definition of that property were identified in the included studies. This does not imply that the property necessarily applies to the instrument or that it was not assessed.

The evidence base is also characterized by considerable heterogeneity. Instruments were developed and/or tested in diverse countries, healthcare systems, and professional groups, which means contextual factors, service models, digital infrastructure, regulatory requirements, language and cultural nuances may have shaped item content, psychometric properties and performance. Applicability beyond the original context therefore remains uncertain unless explicit adaptation and validation work has been reported. Finally, while the search aimed to be comprehensive, relevant instruments may have been missed due to limitations such as to studies published in English, the time frame (between 2015 and the first half of 2025), the exclusion of review-type articles; combined with indexing and keyword variation or limited reporting in the grey literature. Moreover, given the rapid pace of digital innovation, some instruments may have been updated or newly developed since the time of data collection.

## 5 Conclusion

Instruments designed to evaluate multiple dimensions of digital health competence were specifically targeted resulting in a more comprehensive and nuanced overview of available measurement instruments in this field, while simultaneously highlighting the limited and uneven evidence reported for their psychometric validation.

These findings are consistent with the initial premise that digital competence extends beyond discrete technical tasks, reflecting an ongoing effort to define and measure it holistically across roles and settings. This approach also clarifies which competency areas have received the most attention in recent years. Knowing what is being assessed makes it possible to identify current competency levels and to design focused, well-targeted, and methodologically robust education and training that address verified gaps, strengthen practice, and support the digital transition across healthcare.

This mapping also clarifies the development pathways and reported psychometric properties of existing instruments, guiding future studies toward refinement of existing instruments or the design of new ones. Although any instrument should be validated for its specific sample and setting, the availability of psychometrically validated instruments intentionally designed to span multiple professional groups and care environments helps prioritize efforts, supports comparability, and encourages uptake.

At the same time, this is a rapidly evolving field: technologies, workflows, and policies continue to change, making a single fixed definition difficult. Even so, a set of dimensions consistently recurs and overlaps across digital health areas, professions and settings, providing a stable backbone for defining and developing digital health competence.

These findings also contribute meaningfully to the advancement of the digital public health field by clarifying a generalizable core set of competencies that can serve as a foundation for a standardized DiPH-oriented framework for the public health workforce. In addition, the emerging shift toward assessing determinants of digital adoption, such as organizational support, training opportunities, and peer influence, offers a complementary perspective for designing more targeted educational interventions. This approach not only enhances the precision of competency development strategies but also helps to uncover system level barriers that may hinder digital transformation efforts. Taken together, this mapping sharpens research priorities, provides a clear educational and training roadmap essential for a sustainable digital transformation of health systems, and makes a substantive contribution to advancing digital health competency assessment.
