# Supplementary material for: Instruments to assess the digital health competencies of healthcare professionals: a scoping review
Source: Front Public Health. 2026 Jan 5;13:1726452. doi: 10.3389/fpubh.2025.1726452 (PMC12812613; doi:10.3389/fpubh.2025.1726452)
Supplement: Supplementary file 1 [file Table_1.docx]

Supplementary Material

# Supplementary Tables

- 1. **Supplementary Table S1.** Search Strategy.

| **Database** | **Date of search** | **Keywords** | **Filters applied** | **Results retrieved** |
| --- | --- | --- | --- | --- |
| Pubmed #1 | 26/06/2025 | ("Digital Health"[Mesh] OR "Digital Technology"[Mesh] OR "Telemedicine"[Mesh] OR telehealth OR eHealth OR "virtual medicine" OR mHealth OR "mobile health" OR telecare OR "tele-care" OR "tele care" OR "tele intensive care" OR "Distance Counseling"[Mesh] OR "Remote Consultation"[Mesh]) AND "Professional Competence"[Mesh] AND ("Health Personnel"[Mesh] OR "Nurses"[Mesh] OR "Physicians"[Mesh] OR "Caregivers"[Mesh] OR "Dental Staff"[Mesh] OR "Nutritionists"[Mesh] OR "Pharmacists"[Mesh] OR "Physical Therapists"[Mesh] OR "Psychotherapists"[Mesh] OR "Medical Laboratory Personnel"[Mesh] OR "healthcare worker" OR "healthcare workers") | From 1/1/2015 to 26/06/2025 | 193 |
| Pubmed #2 |  | ("Digital Health"[Mesh] OR "Digital Technology"[Mesh] OR "Telemedicine"[Mesh] OR "Remote Consultation"[Mesh] OR telemedicine OR telehealth OR "remote consultation") AND ("Professional Competence"[Mesh] OR awareness OR knowledge OR attitude OR skills OR "Health Knowledge, Attitudes, Practice"[Mesh] OR "Attitude of Health Personnel"[Mesh]) AND ("Health Personnel"[Mesh] OR" health professional" OR "health personnel") AND (faculty OR "teaching hospital" OR "medical college") | From 01/01/2016 to 31/12/2016 | 30 |
| Pubmed #3 |  | ("Digital Health"[Mesh] OR "Digital Technology"[Mesh] OR "Telemedicine"[Mesh] OR "Remote Consultation"[Mesh] OR "Nursing Informatics"[Mesh] OR "Medical Informatics"[Mesh]) AND "Professional Competence"[Mesh] AND "Surveys and Questionnaires"[Mesh] | From 01/01/2018 to 31/12/2018 | 30 |
| Pubmed #4 |  | ("Nursing Informatics"[Mesh] OR "Medical Informatics"[Mesh]) AND ("Surveys and Questionnaires"[Mesh] OR "Psychometrics"[Mesh]) AND "Reproducibility of Results"[Mesh] | From 01/01/2023 to 31/12/2023 | 32 |
| Pubmed #5 |  | ("Digital Health"[Mesh] OR "Digital Technology"[Mesh] OR "Telemedicine"[Mesh] OR "Remote Consultation"[Mesh)] OR ("Health Information Systems"[Mesh] OR "Informatics"[Mesh] OR "Medical Informatics"[Mesh] OR "Nursing Informatics"[Mesh])) AND "Clinical Competence"[Mesh] AND "Surveys and Questionnaires"[Mesh] AND ("Health Personnel"[Mesh] OR "Nurses"[Mesh]) | From 01/01/2023 to 31/12/2023 | 5 |
| Pubmed#6 |  | ("Digital Health"[Mesh] OR digital) AND ("Professional Competence"[Mesh] OR "Clinical Competence"[Mesh] OR "competence") AND ("Nurses"[Mesh] OR nurse OR nurses OR nursing) AND ("Psychometrics"[Mesh] OR "Surveys and Questionnaires"[Mesh] OR questionnaire) AND ("Reproducibility of Results"[Mesh] OR validation) | From 01/01/2024 to 31/12/2024 | 6 |
| Scopus | 26/06/2025 | ("Digital Health" OR "Digital Technology" OR "Telemedicine" OR telehealth OR ehealth OR "virtual medicine" OR mhealth OR "mobile health" OR telecare OR "tele-care" OR "tele care" OR "tele intensive care" OR "Distance Counseling" OR "Remote Consultation" AND "Professional Competence" AND "Health Personnel") | 2015-2025 | 153 |
| Web of Science | 26/06/2025 | ("Digital Health" OR "Digital Technology" OR "Telemedicine" OR telehealth OR ehealth OR "virtual medicine" OR mhealth OR "mobile health" OR telecare OR "tele intensive Care" OR "Distance Counseling" OR "Remote Consultation") AND "Professional Competence" AND ("Health Personnel" OR doctors OR "Physicians" OR "Nurses" OR "Caregivers" OR "Healthcare worker" OR health professional) | From 01/01/2015 to 26/06/2025 | 25 |
